# Supplementary material for: Overexpression of ZmSTOP1-A Enhances Aluminum Tolerance in Arabidopsis by Stimulating Organic Acid Secretion and Reactive Oxygen Species Scavenging
Source: Int J Mol Sci. 2023 Oct 27;24(21):15669. doi: 10.3390/ijms242115669 (PMC10649276; doi:10.3390/ijms242115669)
Supplement: Supplementary file 1 [file ijms-24-15669-s001.zip › ijms-2634576-supplementary.pdf]

Supplemental data

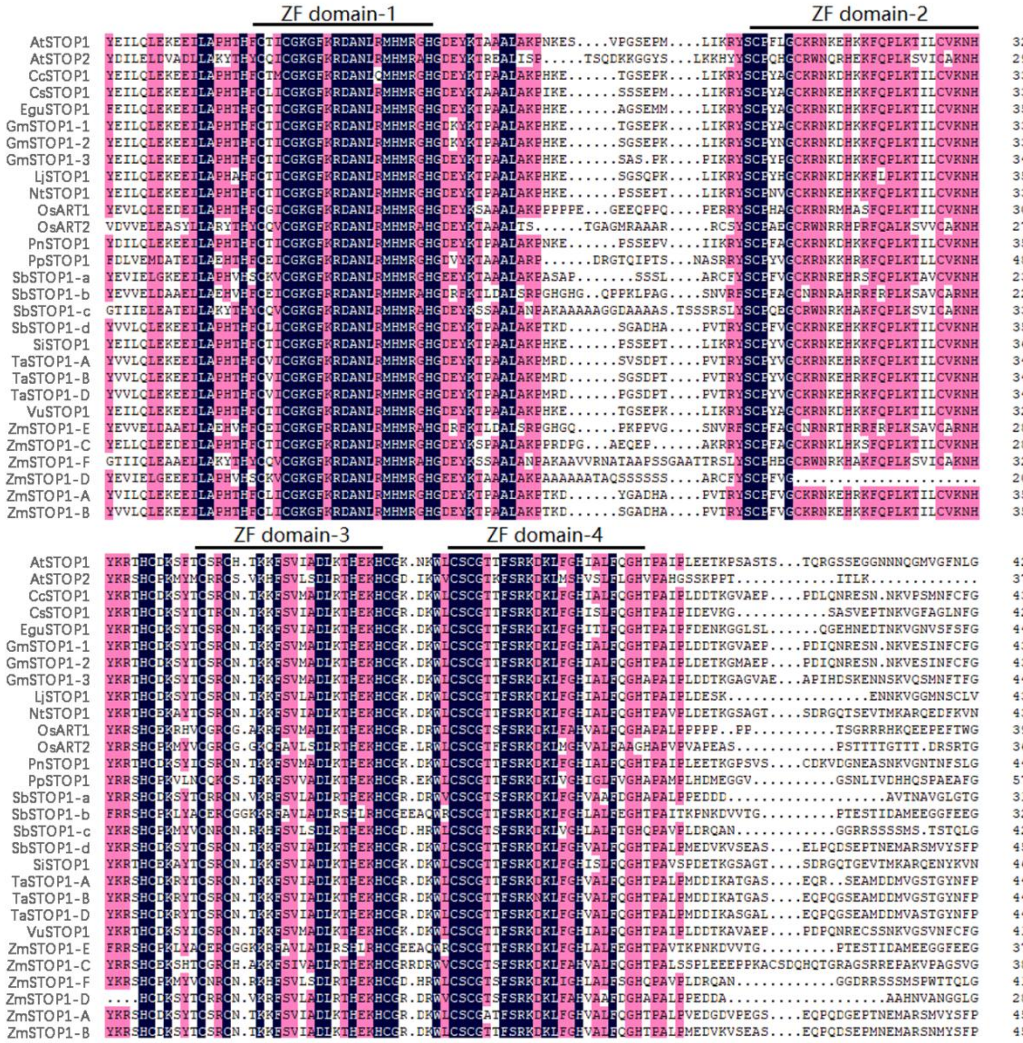

Figure S1. Sequence alignment of ZmSTOP1-A with other STOP1-like transcription factors. Black shading indicates identical residues. Black lines mark conserved Cys and His residues of C2H2 motifs. Aligned STOP1 proteins include representatives from *Arabidopsis* (AtSTOP1, NP_174697.1; AtSTOP2, At5g22890), *Cajanus cajan* (CcSTOP1, MF377546), *Camellia sinensis* (CsSTOP1, BAN67815.1), *Eucalyptus* (EguSTOP1, BAO56822.1), *Glycine max* (GmSTOP1-1, XP_006588359.1; GmSTOP1-2, XP_006598713.1; GmSTOP1-3, XP_014628358.1), *Lotus japonicas* (LjSTOP1, BAN67817.1), *Nicotiana tabacum* (NtSTOP1, AB811781), *Oryza sativa* (OsART1, AB379846; OsART2, Os04g0165200), *Populus nigra* (PnSTOP1, BAN67813.1), *Physcomitrella patens* (PpSTOP1, BAN67814.1), *Sweet sorghum* (SbSTOP1a, Sb01g001950.1; SbSTOP1b, Sb04g023670.1; SbSTOP1c, Sb07g023890.1 and SbSTOP1d, Sb03g041170.1), *Triticum aestivum* (TaSTOP1-A, AGS15201.1; TaSTOP1-B, AGS15202.1; TaSTOP1-D, AGS15195.1) and *Vigna umbellata* (VuSTOP1, KP637172).

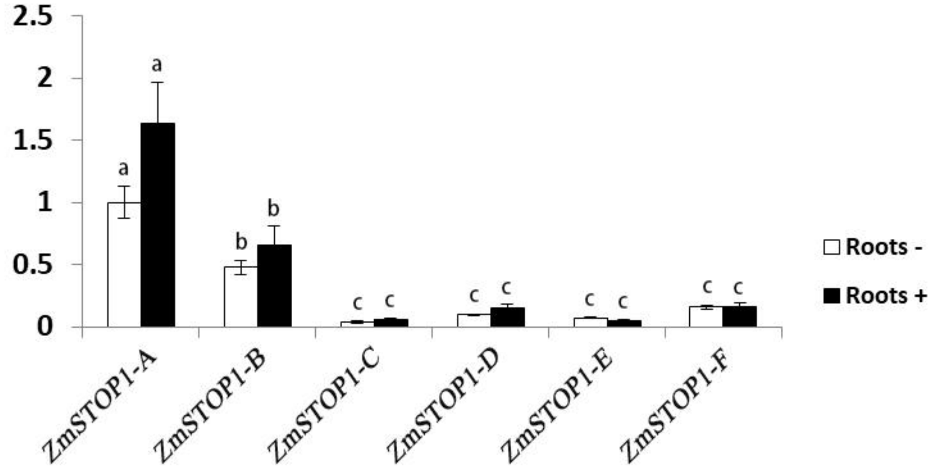

Figure S2. Relative expression of *ZmSTOP1-like* gene in maize seedling roots with 0 or 222 μM Al [$KAl(SO_4)_2$]. Means and SD (n=3) are shown. Different letters indicate significant difference (Tukey's test, $P < 0.05$).

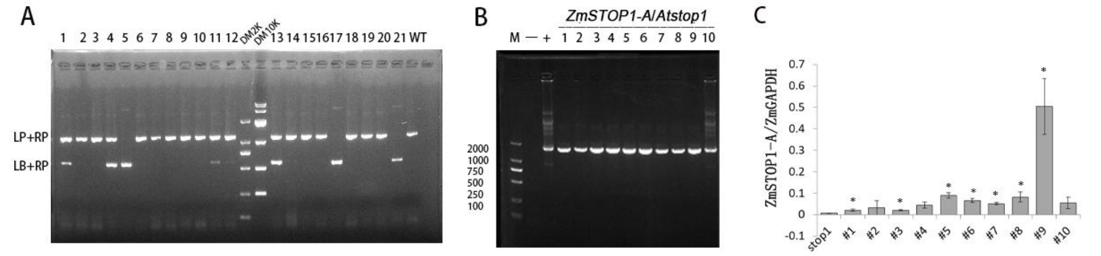

Figure S3. Screening of *Atstop1* mutant, *ZmSTOP1-A/Atstop1* plants and *ZmSTOP1-A* expression in *Atstop1*. (A) PCR screening fo homozygous *Atstop1*(SALK_114108) using RP, LB and LP primers. (B) PCR detection of *ZmSTOP1-A* in transgenic *ZmSTOP1-A/Atstop1*Arabidopsis. (C) Relative expression of *ZmSTOP1-A* detected by RT-PCR. Asterisks indicate significant differences in comparison with *Atstop1* (Tukey's test, $P < 0.05$).

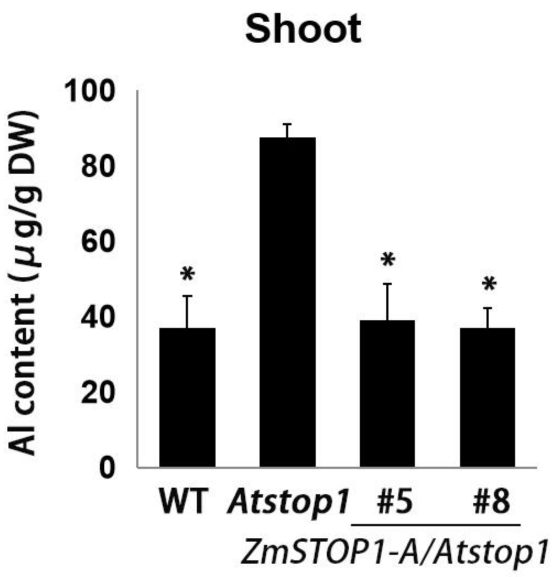

Figure S4. Al contents in shoots of WT, *Atstop1* and *ZmSTOP1-A/Atstop1* plants after 24 h exposure in 20 μM $AlCl_3$ (pH 4.9). Values are mean ± SD ($n \geq 10$). Asterisks indicate significant difference compared to *Atstop1* (Tukey's test, $P < 0.05$).

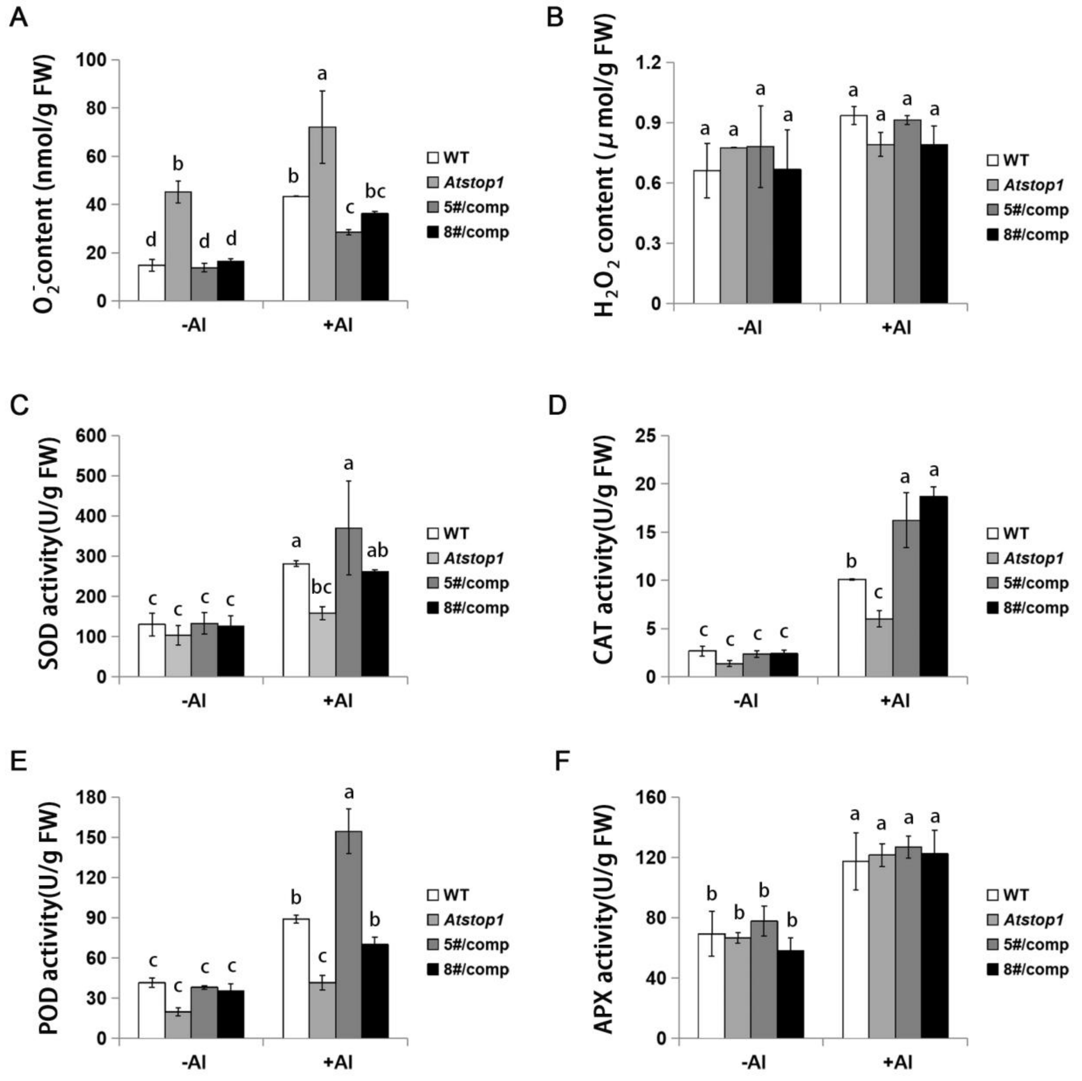

Figure S5. Effects of Al toxicity on reactive oxygen species in WT, *Atstop1*, and *ZmSTOP1-A/Atstop1* shoots. Superoxide anion (O_2^-) (A) and hydrogen peroxide (H_2O_2) (B), activity of SOD (C), CAT (D), POD (E) and APX (F) in shoots after 24 h exposure in 20 μM $AlCl_3$ (pH 4.9). Values are mean ± SD (n ≥ 10) of three experiments. Different letters indicate significant difference (Tukey's test, $P < 0.05$).

Table S1. The primer used in this study

**Names**	**Primer sequences**	**purposes**
ZmSTOP1-A_F	GCATACTGAAGCAGAACTCG	Cloning of ZmSTOP1-A CDS
ZmSTOP1-A_R	AATGACCACAAACGGAGG	
ZmSTOP1-A:GFP_F	GGGGTACCATGGAAGGCGGGATGTCA	Inserted into vector pCAMBIA2300:GFP
ZmSTOP1-A:GFP_R	GCTCTAGAGCTATCACCGTTCTGCTG	
ZmSTOP1-A -CPB_F	GCTCTAGAGCCGAAATGGAAGGCGGGAT	Inserted into vector pCAMBIA:35S-Bar
ZmSTOP1-A-CPB_R	CGGGATCCCGTCACTAATTGGATCCTTA	
CPB-35S-F	GTGGAAAAGGAAGGTGGCTC	PCR detection of overexpressed Arabiaopsis
SALK_11410STOP1-B_LP	AACATCAGTCGCGGAAACTC	PCR detection of *Atstop1* mutant
SALK_11410STOP1-B_RP	GGAGCAGAAGATTCACCAGC	
pZmSTOP1-A_F	GAGTTGTCTTTGGACTTGGG	Cloning of *ZmSTOP1-A* promoter
pZmSTOP1-A_R	GCTTCTACTATCCCGTTTGG	
pZmSTOP1-A:GUS_F	CCCAAGCTTGAGTTGTCTTTGGACTTGGG	Inserted into vector pCAMBIA3301:GUS
pZmSTOP1-A:GUS_R	CATGCCATGGGCTTCTACTATCCCGTTTGG	
qZmGAPDH_F	CCATCACTGCCACACAGAAAAC	Reference gene for qRT-PCR analysis
qZmGAPDH_R	AGGAACACGGAAGGACATACCAG	
qZmSTOP1-A_F	CGTGACAAGTGGCTCTGTT	qRT-PCR analysis of *ZmSTOP1-A* expression
qZmSTOP1-A_R	TGAGGCTGCTCTGAACCTT	
qZm00001d012260_F	CAGAGCAACCTCAGGACAGT	qRT-PCR analysis of *Zm00001d012260* expression
qZm00001d012260_R	CGAGAAATAACCACGCACAT	
qZm00001d034783_F	CCACACGCCAGCAGTTACTA	qRT-PCR analysis of *Zm00001d034783*expression
qZm00001d034783_R	GAATGCCTTCTTCAAATCCC	
qZm00001d023558_F	GACCATCCTGTGCGTGAAGA	qRT-PCR analysis of *Zm00001d023558* expression
qZm00001d023558_R	AGAGCACTGTTCTCCCCTGA	
qAtALMT1_F	CTAGCAAGATTGTCGGGTGC	qRT-PCR analysis of *AtALMT1* expression

qAtALMT1_R	ACAACGATATCAGCGCGAAC	
qAtMATE_F	GTAGCTGGCCAGGCAATACTAGC	qRT-PCR analysis of *AtMATE* expression
qAtMATE_R	GCCACAAACGGAAGTCCTATGC	
qAtALS3_F	CGTATCTCTTCATGGTCTCTGTCG	qRT-PCR analysis of *AtALS3* expression
qAtALS3_R	GTAACTCCGGTGACGGTCATG	
qAtSTOP2_F	GGCACGAGAAGTTTCAACCG	qRT-PCR analysis of *AtSTOP2* expression
qAtSTOP2_R	AGTCCCACAAGAGCAAACCC	
qAtGDH1_F	GAAGGTGAACGATGAGCTAAAGACT	qRT-PCR analysis of *AtGDH1*expression
qAtGDH1_R	GCGTCTTCTAGTACTTGTAAACACA	
qAtGDH2_F	AGGAGTGACAGTGAGTTACTTCG	qRT-PCR analysis of *AtGDH2* expression
qAtGDH2_R	TGGCTCGAGCGACTCGGTTA	
qAtCIPK23_F	CAGTCGACATAAGCTGTTTACTTTT	qRT-PCR analysis of *AtCIPK23* expression
qAtCIPK23_R	GAATCTCAGCGTCCGACAATATAGAA	
qAtNIP1;2-F	GGTTCGATATACTGATAAGCCA	qRT-PCR analysis of *AtNIP1;2* expression
qAtNIP1;2-R	GATACAACTTAACCTCCGATGAC	
qAtACT2_F	GCTGGATTCTGGTGATGGT	Reference gene for qRT-PCR analysis
qAtACT2_R	GCTCTGCTGTTGTGGTGAA	
ZmSTOP1-A:GUS_F	CGGGATCCATGGAAGGCGGGATGTCA	Inserted into vector pBI221:35S-GUS
ZmSTOP1-A:GUS_R	CGAGCTCTCAGCTATCACCGTTCTG	
pZmMATE1:LUC_F	CCAGGCTTTACACTTTATGCTTC	Inserted into vector pBI221:LUC
pZmMATE1:LUC_R	TCTCCAGCGGTTCCATCTTC	
ZmSTOP1-A-AD/BK_F	GGAATTCGCATACTGAAGCAGAACTCG	Inserted into vector pGADT7 or pGBKT7
ZmSTOP1-A-AD/BK_R	CGGGATCCAATGACCACAAACGGAGG	
pM1-1_F	CCCAAGCTTAGGTGGCCTCTTGGAGTT	Inserted into vector pAbAi
pM1-1_R	GGGGTACCGCCGACCTTCTTCAGAGC	
pM1-2_F	CCCAAGCTTAAGGCCCATGCCAAGCAG	Inserted into vector pAbAi

pM1-2_R	GGGGTACCTGGGACCATATTCTTTGA	
pM1-3_F	CCCAAGCTTGAGGACGTTGTTGGAGTT	Inserted into vector pAbAi
pM1-3_R	GGGGTACCCCTCCGGCTCCCCGAGTCC	
pM1-4_F	GGGGTACCGCCACGGGGGAAGGCAAT	Inserted into vector pAbAi
pM1-4_R	CCCAAGCTTGGAAGCGGCGACCGCGCG	

Table S2. Summary of STOP1-like genes involved in Al and/or low pH tolerance

**Species**	**Gene**	**Transformation host**	**Transformation method**	**Aluminum**	**Low pH**	**Regulatory Genes （comp *Atstop1*)**
Arabidopsis	*AtSTOP1*	A.thaliana	Mut/Comp	(QRT-PCR)-; (RRG)+	(QRT-PCR)-; (RRG)+	Al: *AtALMT1, AtALS3*
	AtSTOP2	A.thaliana	RNAi	(QRT-PCR)-; (RRG)+	(QRT-PCR)-; (RRG)+	Al: *AtMATE，AtALS3*
		Atstop1	Comp	(RRG)Slightly	(RRG)+	Low pH: *AtPGIP1/2 ; AtCIPK23*
Rice	*OsART1*	O.sativa	Mut/comp	(QRT-PCR)+; (RE)+	(QRT-PCR)-; (RE)-	/
	OsART2	O.sativa	Mut	(QRT-PCR)+; (RRG)+	(QRT-PCR)-; (RRG)-	/
Wheat	*TaSTOP1-A*	/	/	(QRT-PCR)+	(QRT-PCR)+	/
	TaSTOP1-B	/	/	(QRT-PCR)-	(QRT-PCR)+	/
	TaSTOP1-D	/	/	(QRT-PCR)-	(QRT-PCR)-	/
Tobacco	*NtSTOP1*	N.tabacum	RNAi	(QRT-PCR)-; (RRG)+	(RRG)+	Al: *AtPLT3, AtALS3, AtMATE, AtGDH1, AtALMT1*; Low pH: *AtPGIP1, AtCIPK23, AtSTOP2*
		Atstop1	Comp	(RRG)Slightly	(RRG)+	
Lotus	*LjSTOP1*	*Atstop1*	Comp	(RRG)Slightly	(RRG)+	Al: *AtPLT3, AtGDH1, AtALMT1*; Low pH: *AtPGIP1, AtCIPK23, AtSTOP2*
Tea	*CsSTOP1*	*Atstop1*	Comp	(RRG)-	(RRG)+	Al: *AtALMT1*; Low pH: *AtPGIP1, AtCIPK23,AtSTOP2*
Black poplar	*PnSTOP1*	*Atstop1*	Comp	(RRG)Slightly	(RRG)+	Al: *AtALMT1, AtGDH1, AtPLT3*; Low pH: *AtPGIP1, AtCIPK23,AtSTOP2*
Moss	*PpSTOP1*	P.patens	RNAi	(Growth)+	(Growth)-	Al: *AtALMT1, AtMATE, AtGDH1, AtPLT3, AtALS3*;
		Atstop1	Comp	(RRG)+	(RRG)+	Low pH: *AtPGIP1, AtCIPK23, AtSTOP2*

Eucalyptus	*EguSTOP1*	*Eucalyptus*	RNAi	(QRT-PCR)-;<br>(RE)+	(QRT-PCR)-;<br>(RE)+	Al: *AtALS3, AtMATE*;<br>Low pH: *AtPGIP1, AtCIPK23, AtSTOP2*
		Atstop1	Comp	(RRG)-	(RRG)+	
Rice bean	*VuSTOP1*	*Atstop1*	Comp	(QRT-PCR)+;<br>(RE)Slightly	(QRT-PCR)+;<br>(RE)+	Al: *AtALS3, AtMATE*;<br>Low pH: *AtPGIP1, AtCIPK23, AtSTOP2, AtGDH1*
pigeonpea	*CcSTOP1*	C.cajan	RNAi	(QRT-PCR)-	/	/
Sweet sorghum	*SbSTOP1-a*	/	/	(QRT-PCR)+	/	/
	SbSTOP1-b	/	/	(QRT-PCR)+	/	/
	SbSTOP1-c	/	/	(QRT-PCR)+	/	/
	SbSTOP1-d	*Atstop1*	Comp	(QRT-PCR)+;<br>(RE)+	/	Al: *AtMATE* , *AtALS3*;<br>Low pH: *AtPGIP1/2* ; *AtCIPK23*
Soybean	*GmSTOP1-1*	*Atstop1*	Comp	(QRT-PCR)+;<br>(RE)Slightly	(RE)+	Al: *AtPM1, AtTDT, AtNADP-ME2, AtMATE*;<br>Low pH: *AtGDH1/2, GABA-T, AtNADP-ME2*
	GmSTOP1-2	*Atstop1*	Comp	(QRT-PCR)-;<br>(RE)-	(RE)+	Low pH: *AtGDH1/2, GABA-T, AtNADP-ME2*
	GmSTOP1-3	*Atstop1*	Comp	(QRT-PCR)+;<br>(RE)Slightly	(RE)+	Al: *AtPM1, AtTDT, AtNADP-ME2, AtMATE*;<br>Low pH: *AtGDH1/2, GABA-T, AtNADP-ME2*
Cotton	*GhSTOP1*	G.hirsutum	RNAi	(RRG)+	(RRG)+	Al: *AtMATE* , *AtALS3*;<br>Low pH: *AtPGIP1/2* ; *AtCIPK23*
Barley	*HvAF1*	H. vulgare	RNAi	(QRT-PCR)-	/	/
Rye	*ScSTOP1*	*Atstop1*	Comp	(RE)+	(RE)+	Al: *ScALMT1*

Exegesis: RE: Root elongation; RRG: Relative Root Growth; +: Respond to Al toxicity or low pH; -: Not respond to Al toxicity or low pH.
